# THE RELATIONSHIP BETWEEN SELF-REPORTED QUALITY OF LIFE AND SMARTPHONE GPS DATA

**DOI:** 10.1093/geroni/igad104.2822

**Published:** 2023-12-21

**Authors:** Kyungmi Lee, Andres Azuero, Sidharth Kumar, Frank Puga, George Demiris, Christine Ritchie, Alexi A Wright, J Nicholas Dionne-Odom

**Affiliations:** Case Western Reserve University, Cleveland, Ohio, United States; University of Alabama at Birmingham, Birmingham, Alabama, United States; University of Alabama at Birmingham, Birmingham, Alabama, United States; University of Alabama at Birmingham, Birmingham, Alabama, United States; University of Pennsylvania, Philadelphia, Pennsylvania, United States; Massachusetts General Hospital, Boston, Massachusetts, United States; Dana-Farber Cancer Institute, Boston, Massachusetts, United States; University of Alabama at Birmingham, Birmingham, Alabama, United States

## Abstract

Patients with advanced cancer and their family caregivers often experience poor quality of life. Self-reported measures are commonly used to quantify quality of life of family caregivers but may have limitations such as recall and social desirability bias. Variables derived from passively obtained smartphone GPS data are a novel approach to measuring quality of life that may overcome these limitations, and enable detection of early signs of deteriorating mental and physical health. The purpose of this study was to examine the temporal directionality of the correlations between self-reported quality of life (i.e., PROMIS Global 10: physical and mental health domains) at 3 timepoints and passively acquired smartphone GPS data among 7 family caregivers and 4 advanced cancer patients over a 12-week period. We extracted variables (i.e., total distance, time spent at home, transition time, and number of significant locations) with algorithms applied to the raw GPS sensor data. Uncertainty measures for the correlation estimates were computed as uncorrected 95% Bootstrap confidence intervals. We found a medium-to-large correlation between quality of life and the daily smartphone GPS data averaged by week in certain time periods (e.g., temporal correlations between physical health and transition time for 0–6 weeks; r’s ranging from 0.30 to 0.53). Additionally, we found a medium-to-large correlation between quality of life and within-person variability (standard deviation) in daily smartphone GPS data. Our findings indicate that variation in within- and between-person behavior patterns captured by GPS data may signal potential changes in individuals’ physical and mental health outcomes.

